# The Annual Conventions

**Published:** 1894-07

**Authors:** 


### THE ANNUAL CONVENTIONS.

The year 1893 was devoted almost entirely, in this country, to the support of the World's Columbian Dental Congress, with the result that the two principal conventions were held over to the present year.

The dentists of the United States should, therefore, show additional energy in support of these meetings, and every effort should be made to make them worthy the increased experience in the work of the profession.

It is not probable that any change in the mode of conducting these annual gatherings will speedily be adopted, and until something better presents it is the duty of all interested to be present and take an active part in their deliberations.

The American Dental Association will meet at Old Point Comfort, Va., August 7, and it is presumed that the Southern Dental Association will also convene at that place, though at this writing we have not received the official call for that meeting.

The National Association of Dental Faculties and National Association of Dental Examiners will, without doubt, meet at the same time and place.

The American Dental Society of Europe, having omitted its meeting last year, will hold its nineteenth convention this year at Geneva, Switzerland, August 6, 7, and 8. Members of the profession everywhere are cordially invited to be present.

Many of the State organizations meet in July, claiming special attention, and will be, from present indications, well attended.

